# Cardiovascular Autonomic Response to Amlodipine in Primary Hypertension

**DOI:** 10.5402/2012/832183

**Published:** 2012-06-25

**Authors:** Youssouf Radjab, Souad Aboudrar, Fatima Zahra Milouk, Hanan Rkain, Mustapha EL Bakkali, Taoufiq Dakka, Leslie Coghlan, Halima Benjelloun

**Affiliations:** ^1^Physiology Laboratory, School of Medicine and Pharmacy, University Mohammed V Souissi, Rabat 6203, Morocco; ^2^Unit of Cardiology A, Ibn Sina University Hospital, Rabat 10000, Morocco; ^3^School of Science and Engineering, Al Akhawayn University, Ifrane 5300, Morocco

## Abstract

Sympathetic hyperactivity may be involved in primary hypertension. The purpose of this study was to evaluate both sympathetic and vagal activity responses in patients receiving amlodipine as antihypertensive agent. *Patients and Methods*. This prospective study included a group of primary hypertensive patients (*N* = 32, mean age 54.6 ± 7.6 years). The cardiovascular autonomic tests performed in this group, before and after 3 months of daily oral administration of amlodipine, included deep breathing, hand-grip, and mental stress tests. Statistical analysis was done using the Student's *t*-test. *Results*. Cardiovascular autonomic reflexes responses before and after 3 months of amlodipine oral administration were as follows: the mental stress test stimulation method produced a central alpha adrenergic response of 23.9 ± 8.7% versus 11.2 ± 2.0% (*P* < 0.05), a central beta sympathetic response of 16.7 ± 9.2% versus 10.4 ± 1.3% (*P* < 0.05), a blood pressure increase in response to hand grip test of 20.5 ± 7.3% versus 10.7 ± 2.4% (*P* < 0.05), vagal response to deep breathing test was 21.2 ± 6.5% versus 30.8 ± 2.9%, (*P* < 0.05). *Conclusion*. The results attest that amlodipine may have an anti-sympathetic effect.

## 1. Introduction

In most cases, hypertension (HT) is primary. The etiopathogeny of HT in general remains an enigma. The central nervous system plays an important part in its acute form and even in its chronic form [1, 2]. Indeed, sympathetic nerve hyperactivity is involved in primary HT [3, 4]. Because of its cardiac and vascular risk, it is an important concern in term of morbimortality [5–7]. The autonomous nervous system (ANS) intervenes in the control of the blood pressure (BP) and the heart rate (HR). Many classes of drugs have proved to be reliable in the treatment of primary hypertension. But in spite of the discovery of new therapeutic molecules, the cardiovascular diseases represent the first cause of mortality in the developed countries. Consequently, research on the physiopathology of HT is of interest for the development of effective therapeutic agents.

Amlodipine, a calcium antagonist, has proved its effectiveness in the treatment of primary hypertension in monotherapy and in association with other antihypertensive drugs [8]. Naturally, it is interesting and important to understand the mechanism of action of amlodipine. The harmful effect of sympathetic nerve hyperactivity is considered currently as a cardiovascular risk factor [9, 10]. An antihypertensive treatment which reduces sympathetic nerve activity would be of an unquestionable benefit. Furthermore, the vagal system is recognized as being a protective system, not only in hypertensives but also in all subjects with cardiovascular risk factors such as diabetes, and ischemic cardiomyopathy. Therefore, it is interesting to study if amlodipine reduces sympathetic nerve activity and/or increases vagal activity thus inducing a shift in sympathovagal balance.

The objective of this work was to compare the cardiovascular autonomic reflexes in hypertensive patients before and after 3 months of treatment with amlodipine. 

## 2. Patients and Methods

This is a prospective study comparing two cardiac autonomic evaluations: pre- and posttherapeutic, carried out in the unit of exploration of the ANS, service of cardiology “A” at the University hospital Ibn Sina. The patients were referred by hypertension specialist physicians. 

The study was approved by the Ibn Sina ethical committee after a thorough analysis. A written consent form was obtained from each patient before the tests. Each patient completed also a form recording the presence or the absence of functional signs.

**Inclusion Criteria**

Patients with BP equal or higher than 140/90 mm Hg, mild to moderate primary HT, not complicated and not yet treated.

**Exclusion Criteria**

Severe HT, secondary or complicated HT, or being under any antihypertensive treatment. Also excluded from this study were patients having over-sensitiveness to the molecule, patients breast feeding, or in pregnancy.

All patients received a three month treatment of 5 mg daily oral administration of amlodipine after the first cardiovascular autonomic testing. After this 3 month period, the second cardiac autonomic testing was performed.

### 2.1. Description of the Tests

The patients were initially placed in a calm environment, in supine position for 30 minutes. Then monitoring of BP, using a Dynamap (Critikon, 1846 SXP) and of HR (screen of posting LCD CS 503 E, HELLIGE, EK 512 E) was done. All the tests were carried out in the morning at fasting and under no hypertensive medication.

### 2.2. The Deep Breathing Test (DB)

This test analyzes the vagal response [11, 12]. The respiratory frequency has an influence on the variation of RR interval on the EKG. The procedure was the following: the patient breathes deeply at a frequency of six breaths for one minute [13]. It makes it possible to evaluate the vagal activity which is expressed as a percentage: (RR_maximal_ − RR_minimal_/RR_minimal_) × 100. 

### 2.3. The Isometric Contraction or Hand Grip Test (HG)

During three minutes the patient performs a manual pressure of 50% of the maximum with assistance of a dynamometer. The muscular contraction involves a rise in BP related to an increase of sympathetic nerve activity at the muscular level that is effort-dependent and time-dependent [14, 15]. The peripheral alpha sympathetic nerve response is given by the increase of the BP.

(1) Peripheral  sympathetic  alpha  activity  (in  %)=(BPafter  the  test−BPbefore  the  testBPbefore  the  test)×100.

### 2.4. The Mental Stress Test

The patient performs mental arithmetic calculations by removing the number 7 successively from 200. The result is an increase in BP and in HR by activation of the central sympathetic nerve [13]. 

In mental stress, the central sympathetic nerves activities “*α*” was evaluated by measuring the variations of BP as bellow [14, 15]:

(2) Central  sympathetic  response  alpha(alpha  SC)=(BPafter  stimulation−BPbefore  stimulationBPbefore  stimulation)×100.

The central sympathetic nerves activities “*β*” was evaluated by measuring the variations of HR as bellow [14, 15]:

(3) Central  sympathetic  response  beta  (beta  SC)=(HRafter  stimulation−HRbefore  stimulationHRbefore  stimulation)×100.

### 2.5. Statistical Analysis

The tests selected for the comparisons between the 2 studies were the following: mental stress for the study of the alpha and beta central sympathetic nerve activities; the hand-grip of 3 min for the study of the alpha peripheral sympathetic nerve activity and the deep breathing test for the study of the vagal system. The values are expressed as average ± SE. 

The comparison of the results before and after amlodipine treatment was carried out by means of the *t*-test Student apparied. A value *P* < 0.05 was considered significant.

In this study, we considered that the systolic blood pressure in noncomplicated hypertensive patients could be representative for BP variation. Our previous study [3] showed the good correlation with sympathetic assessment.

## 3. Results

The mean age of the patients of the study was of 54.6 ± 7.6 years, with the extreme ranging from 40 to 79 years. 

Basal BP decreased to a significant degree after treatment with amlodipine, reaching a normal value (Table 1). The basal HR did not undergo significant modifications. And the vagal response, as measured by the deep breathing test (DB), underwent a significant rise after treatment (Table 2). 

**Figure 1:**
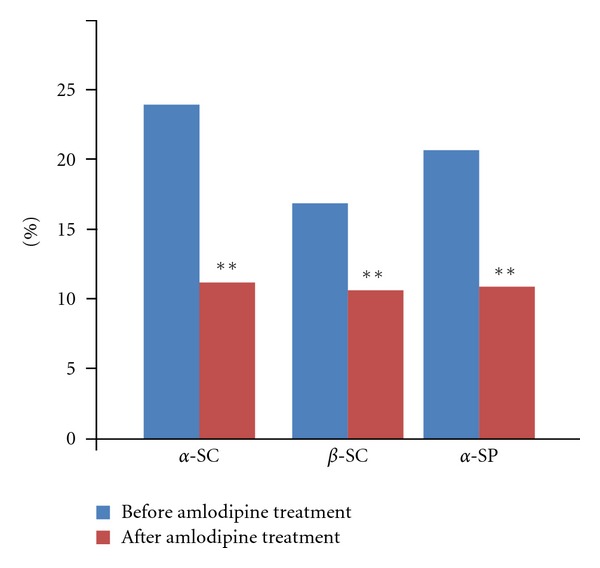
Central sympathetic response alpha (alpha SC), central sympathetic response beta (beta SC) obtained during mental stress, and peripheral sympathetic response alpha (alpha SP) obtained on hand grip test. Parameters were measured before and after treatment by amlodipine and expressed as mean ± SE; **: *P* < 0.01.

The alpha and beta central sympathetic nerve response in the mental stress test (MS) significantly decreased (Table 2, Figure 1). The alpha peripheral sympathetic nerve response in the HG test decreased significantly (Figure 1). 

The undergone tests measured 3 aspects of the sympathetic response (alpha and beta central sympathetic activity, and alpha peripheral sympathetic activity) showing each time significant lowering of this response. This decrease in sympathetic activity was offset by an increase in the vagal response.

## 4. Discussion

Among the examinations necessary to study arterial hypertension, the cardiovascular autonomous nervous system reflexes which include the measurement of the sympathetic and vagal nerve activity is a new approach. In a previous study [3], we compared the autonomic cardiovascular reflexes of a group of hypertensive with a group of normotensive subjects. The sympathetic nerve response was significantly higher and the vagal response was significantly lower in hypertensive patients when compared to normotensive. Thus, the study of the cardiovascular autonomic profile under an antihypertensive treatment is of interest. Since the antihypertensive effect of amlodipine is already proven, the purpose of this study was to analyse the effect of this molecule on the cardiovascular autonomic reflexes. The novelty of this study is to compare both sympathetic and vagal nerve responses to stimulations in patients before and after three months of treatment with Amlodipine, using specifically cardiovascular autonomic tests. The analysis of the results indicates that cardiovascular autonomic reflexes in hypertensives showed a significant reduction of the sympathetic nerve hyperactivity and a significant rise in the vagal response inducing a shift in sympathovagal balance.

The central nervous system (CNS) is involved in the regulation of BP. This regulation is done by several areas of the cerebral cortex like the driving surfaces, the pre-motor area, the fronto-orbital area, and the gyrus cingulaire [1, 16, 17]. The stimulation of the posterior hypothalamus involves an increase in the BP, whereas the stimulation of the anterior hypothalamus involves a reduction in the BP. These various cerebral structures contribute to the regulation of BP via peripheral autonomic nervous system (ANS) regulation [1]. The regulation of the BP and the HR are not only controled by the CNS but also by ANS. This reflex autonomic regulation uses baroreceptors and chemoreceptors, located at the peripheral level [17]. The baroreceptors make it possible to lower or to even remove any variation of pressure due to the change of posture whereas the chemoreceptorss located at the level of the carotid sinus and the aortic arch are stimulated by an increase in the PCO_2_, a reduction in the pH or PO_2_ [18, 19]. These chemoreceptors inhibit the cardio-regulator center and stimulates the vasomotor center, which results in an increase in the HR, a vasoconstriction and consequently an increase in the BP [1]. 

Hypertension represents a serious risk factor for patients. The sympathetic nerve hyperactivity observed in the hypertensive and the inadequate vagal response do not act solely on the cardiac system but reach also the other target bodies of the autonomous nervous system[10, 20, 21]. The excessive rise in the sympathetic nerve activity increases in an acute way the BP while being responsible for cardiac, renal, and vascular stimulation by respectively increasing the cardiac flow, the sodium retention, and vascular resistance with hypertrophy of the vascular smooth muscular cells [10]. The sympathetic nerve hyperactivity is regarded as a risk factor of coronaropathy, cardiac insufficiency, cerebro-vascular accident, and of renal vascular attack [4]. Autonomic dysregulation plays a significant role in the hypertension and acts as coronary risk factor by severe metabolic complications [19]. In the other hand, Schlaich and Krum [22], showed that renal denervation is associated with a substantial and presumably sustained BP reduction. That cofirm link between autonomic nervous system and hypertension control, and assess the role that could be played by exploring the autonomic nervous system of antihypertensive patients before initiating their treatment. 

Milovanović et al. [23] and Sakata et al. [24] showed that amlodipine affected autonomic modulation with a shift to sympathetic hyperactivity, but that was without statical significance for what Milanovic and Sakata et al. have studied about the sympathetic response by biological analysis of plasma rennin activity and norepinephrine concentration which make not possible the comparison of the results.

Interestingly, in this work, we also noted an increase in vagal response after treatment with amlodipine. Under normal conditions and at rest, the vagal brake maintains the HR within a normal value. The mechanism of Starling adjusts beat by beat the ejection and the ventricular filling. Under effort, the HR increases because of the lifting of the vagal brake [16]. A low-vagal activity would be unable to restore the sympathovagal balance to normotensive level [19, 25]. Perhaps the increase in the parasympathetic activity plays a protective role. This protective action would disappear in hypertensive patients. Sympathovagal imbalance may be at the origin of coronary, vascular, and metabolic complications.

The sympathovagal balance studies in essential hypertension before and after antihypertensive drug are becoming a new approach to evaluate hypertension and its treatment [26–32]. 

## 5. Conclusion

To lower the sympathetic nerve response and/or to increase the vagal response is a very valuable therapeutic objective. Cardiovascular complications due to sympathetic nerve hyperactivity could be avoided among these patients, knowing the existence of a sympathetic nerve hyperactivity in the primary hypertension [3, 18]. Thus, it is judicious to study the sympathetic and vagal nerve activities under amlodipine at a group of hypertensive patients. This study indicated that this molecule, in addition to its antihypertensive effect, reduced to a significant degree the sympathetic nerve hyperactivity and increased the vagal activity. 

## Figures and Tables

**Table 1:** Values of blood pressure (BP, mmHg) and heart rate (HR, beats/min) recorded at rest, before, and after amlodipine treatment. Values are expressed as mean ± SE, ^∗∗^: *P* < 0.001.

Test	Basal BP (mmHg)	Basal HR (b/min)
Before amlodipine treatment	152.4 ± 13.6	64.5 ± 8.2
After amlodipine treatment	123.2 ± 8.1^∗∗^	67.5 ± 12.2

**Table 2:** Vagal response on deep breathing test (XDB). Central sympathetic response alpha (alpha SC), central sympathetic response beta (beta SC) obtained during mental stress, and peripheral sympathetic response alpha (alpha SP) obtained on hand grip test. Parameters were measured before and after treatment by amlodipine, and expressed as mean ± SE; ^∗∗^: *P* < 0.01.

Test	XDB (%)	Alpha SC (%)	Beta SC (%)	Alpha SP (%)
Before amlodipine treatment	21.2 ± 6.5	23.9 ± 8.7	16.7 ± 9.2	20.5 ± 7.3
After amlodipine treatment	30.8 ± 2.9^∗∗^	11.2 ± 2.0^∗∗^	10.4 ± 1.3^∗∗^	10.7 ± 2.4^∗∗^
